# Modelling the Effects of Growth and Remodelling on the Density and Structure of Cancellous Bone

**DOI:** 10.1007/s11538-024-01267-3

**Published:** 2024-03-04

**Authors:** Brianna L. Martin, Karen J. Reynolds, Nicola L. Fazzalari, Murk J. Bottema

**Affiliations:** 1 Marine Spatial Ecology Laboratory, School of the Environment, The University of Queensland, Level 5, Goddard Building, St. Lucia, QLD 4072 Australia; 2 Medical Device Research Institute, College of Science and Engineering, Flinders University, Tonsley Campus, 1284 South Rd, Clovelly Park, SA 5042 Australia

**Keywords:** Cancellous bone, Remodelling, Mathematical model, Resorption volume, Formation fraction, Activation frequency

## Abstract

A two-stage model is proposed for investigating remodelling characteristics in bone over time and distance to the growth plate. The first stage comprises a partial differential equation (PDE) for bone density as a function of time and distance from the growth plate. This stage clarifies the contributions to changes in bone density due to remodelling and growth processes and tracks the rate at which new bone emanates from the growth plate. The second stage consists of simulating the remodelling process to determine remodelling characteristics. Implementing the second stage requires the rate at which bone moves away from the growth plate computed during the first stage. The second stage is also needed to confirm that remodelling characteristics predicted by the first stage may be explained by a realistic model for remodelling and to compute activation frequency. The model is demonstrated on microCT scans of tibia of juvenile female rats in three experimental groups: sham-operated control, oestrogen deprived, and oestrogen deprived followed by treatment. Model predictions for changes in bone density and remodelling characteristics agree with the literature. In addition, the model provides new insight into the role of treatment on the density of new bone emanating from the growth plate and provides quantitative descriptions of changes in remodelling characteristics beyond what has been possible to ascertain by experimentation alone.

**Supplementary Information:**

The online version contains supplementary material available at 10.1007/s11538-024-01267-3.

## Introduction

Bone undergoes constant renewal to replace old and damaged bone and to compensate for changes in loading due to age, injury or change in lifestyle. The main mechanism for this change is remodelling. Remodelling takes place locally at individual sites called basic multicellular units (BMU). A highly simplified picture of remodelling is that cells called osteoclasts remove a small amount of bone during a resorption phase and cells called osteoblasts replace bone during the formation phase. The net loss or gain of bone is determined by the amount of bone removed (resorption volume) and the fraction of the resorption volume replaced during formation (formation fraction). The full picture is much more complicated and has been studied extensively. A review of remodelling presenting resorption and formation as coordinated processes instead of separate events appears in Lerner et al. (2019). A recent and comprehensive picture of remodelling viewed as a consequence of a global network of cellular signalling may be found in Bolamperti et al. (2022). Mathematical modelling has played a significant role in understanding remodelling and its consequences. Although a multi-scale understanding, ranging from sub-cellular to full organ, is the eventual goal, model objectives may be viewed as falling into three categories: models to explain the biochemical and cellular interplay at the level of individual BMU; models at the local tissue level; and models at the whole organ level to link understanding of bone loss and remodelling to clinical practice in the form of prosthetic design or planning of care. Some details are provided in the subsequent subsections.

The spatio-temporal changes in bone are further complicated during growth. New bone emerges from the growth plate and moves along the axis of the bone away from the growth plate. Thus at any one location and point in time both the emergence of new bone and remodelling contribute to the dynamics.

### Models within BMU

In 2003, Komarova et al. presented an ordinary differential equation model for total bone mass at a remodelling site as governed by coupled osteoblast and osteoclast cell populations. Cell production is regulated by autocrine and paracrine interactions, based on known effects of cell signalling factors including receptor activator of nuclear $\kappa B$ (RANKL), transforming growth factor $\beta$ (TGF$\beta$), insulin-like growth factors (IGF), and osteoprogeterin (OPG). For simplicity, bone removal was modelled as proportional to the respective cell populations, though some of the above signalling factors, including RANKL and IGF, are known to promote cell survival (Komarova et al. 2003). Other factors promoting osteoclast and oseoblast survival such as receptor activator of nuclear $\kappa$B ligand (RANKL) were not specifically included. In the next year, a model was proposed by Lemaire et al. (2004) which included RANKL and OPG explicitly, the latter being highly expressed in osteoblasts. A subsequent extension of the Komarova model included a term for anabolic treatment, parathyroid hormone (PTH). Duration-dependent PTH treatment was introduced to the system through the osteoblast derived regulation on osteoclast cells (Komarova 2005). The same group produced a model focussing on the dynamics of osteoclast lifecycles (Akchurin et al. 2008) and in 2009 produced a spatial-temporal model for individual remodelling events based on nonlinear PDE (Ryser et al. 2009). Van Oers et al. (2008) published a model used to demonstrate how strain induces the process of remodelling and explain the mechanism of repairing micro-damage in bone.

Also in 2008, Pivonka et al. proposed an extension of the model by Lemaire et al. (2004) to include the rates at which key process in the BMU occur and to allow for more detail regarding the expression of RANKL and OPG (Pivonka et al. 2008). Work was also reported on modelling the action of osteoclasts in producing the physical shape of resorption cavities (Buenzli et al. 2012). The same group produced a comprehensive PDE model for cell development and the refilling process spatially along the axis of the remodelling cavity which was able to replicate patterns of bone tissue apposition observed experimentally (Buenzli et al. 2014). In 2020, Ayati et al. used the Komarova model (Komarova et al. 2003) and a perturbed version of this model to compare remodelling in normal situations with remodelling in the presence of myeloma (Ayati et al. 2010). This work was extended by Peyroteo at al. (2020) to include a 2-dimensional diffusion model for remodelling. The model thus incorporated the spatial distribution of cells in the BMU, albeit in 2 dimensions, whereas the previous models either considered only time or the spatial direction along the axis of the BMU.

Hernandez et al. (2001) modelled the interplay between duration of the various stages of individual remodelling events, bone volume and the rate at which new remodelling events are initiated. The model did not include the biochemical basis for cellular dynamics as did the studies mentioned above and the model did not address the shape of remodelling cavities or the consequences regarding changes in structure.

### Whole Organ Models

Nackenhorst used finite elements (FE) to compute stress in natural femurs and femurs with virtual prosthetics in 1997 (Nackenhorst 1997). By assuming that remodelling would target the regions of stress identified by the FE model, the study predicted shapes and dimensions for improving prosthetic design. No details of the remodelling characteristics or processes within the BMU were incorporated in the model. A 3-dimensional FE model of the femur, coupled with a thermodynamic model for remodelling to study long term consequences of hip replacements, was published in 2014 (Avval et al. 2015). Lerebours et al. (2016) presented a model linking mechanical and biological aspects of bone remodelling to study bone consequent to osteoporosis and mechanical disuse. This multi-scale model considers the biochemical and cellular interactions following (Pivonka et al. 2008) at a scale of 10–20 $\upmu$m, the impact of remodelling on tissue properties at a scale of 2–5 cm and stress at the scale of the entire bone (5–45 cm). The modelling of stress used generalised beam theory and micromechanics (Hellmich et al. 2008; Scheiner et al. 2013). More recently (2022) (Quexada et al. 2022) published a comprehensive model linking cell dynamics adapted from Komarova et al. (2003) with the mechanics model of Nackenhorst (1997). This model was able to produce realistic trabecular structure for bone at equilibrium between resorption and formation (normal bone) and in bone where remodelling dynamics mimic osteoporosis.

### Local Tissue Structure and Remodelling

Modelling studies aimed at improving care or the design of prosthetics naturally focus on targeted remodelling, remodelling initiated to repair damage or compensate for changes in loading. In addition to targeted remodelling, non-targeted remodelling takes place constantly in healthy as well as diseased bone. Remodelling results in a temporary reduction of bone volume as a result of resorption which is eventually restored by formation (Seeman and Martin 2019). However, the balance between resorption and formation as well as the activation frequency (defined as the average number of new remodelling events initiated at a specific site per unit time Parfitt et al. 1987) are influenced by disease and ageing and can lead to bone loss.

Ageing is associated with increases in the number of remodelling per unit time (increased activation frequency), increases in the sizes of resorption cavities and decreases in formation volume so that, at the tissue level, ageing results in bone loss (Vedi et al. 1983; Han et al. 1997; Seeman and Martin 2019. In animal studies, bone loss is often induced in female animals by ovariectomy resulting in oestrogen deprivation. The effects on remodelling are similar to that of ageing (Yoon et al. 2012).

During growth, the local structure of bone is also influenced by the emergence of new bone from the growth plate also known as the physis or epiphyseal plate. In this case, changes in bone density at a fixed distance from the growth plate reflect both new bone and remodelling. This is important in interpreting studies based on young animals and should be taken into account when applying results from studies on human bone from elderly donors to children.

Studies on characterising resorption cavity volumes, formation volumes, activation frequencies and related remodelling characteristics are difficult to perform. Goff et al. (2012) characterised size and shape of resorption cavities in human trabecular bone in 2012. The data comprised micro-computed tomography ($\mu$CT) scans of vertebral cancellous bone from four elderly donors (47–80 years of age). Resorption cavities were detected and measured manually by noting eroded surface profiles in individual cross sections. Three-dimensional rendering was used to obtain a visual image of the cavity. The observer traced the surface of the cavity using a software tool. Local trabecular morphology was then traced manually to obtain local structure and orientation information relative to the resorption cavity. Matheny et al. (2013) studied resorption cavity size in lumbar vertebrae of adult female rats. Labelling with luminescent metabolic labels for bone formation was used to facilitate identification of newly formed bone. Three dimensional measures of resorption cavities were obtained by manual tracing of eroded regions found visually in cross sections. Manual tracing of resorption cavities is labour intensive and so only 8–32 resorption cavities were identified per specimen.

### Scope of the Study

Here a two-stage model is introduced for studying remodelling at the tissue level. Neither the biochemical mechanisms for remodelling are considered nor are the large scale consequences at the whole organ level are considered. Instead the models consider the combined effect of remodelling and the emergence of new bone from the growth plate at the tissue level.

The first stage comprises a partial differential equation (PDE) for the density of bone over time and distance from the growth plate. Solutions to the PDE provide the rate at which new bone moves away from the growth plate allowing locations of remodelling to be tracked over time. This stage also provides information about the *average* bone loss per unit time due to remodelling. However, this model does not provide information on whether the observed bone density patterns are achievable by realistic remodelling parameters. Should this not be the case, then phenomena other than growth and remodelling must play a significant role in driving bone density. The second stage determines remodelling parameters which best explain bone structure changes due to remodelling and demonstrates that, indeed, growth and remodelling suffice to explain the observed densities patterns.

In the second stage, an algorithm for simulating remodelling is applied to a $\mu$CT reconstruction of a sample of bone at an initial time $t_1$. The structure of the sample after simulated remodelling is compared to the structure of the actual sample at a later time $t_2$. The discrepancy between the simulated structure and the actual structure indicates the quality of the simulation. Since the actual bone sample moves away from the growth plate over the time interval $[t_1,t_2]$, the rate at which this happens is needed to extract the the sample at time $t_2$ from the correct location. This rate is provided by the first stage of the model along with other parameters needed to align data at different time points. Details regarding the data samples and time points are provided in Sect. 2.1.

The model requires only $\mu$CT data at multiple time points. The method does not require the use of metabolic labels, manual detection or tracing of resorption cavities or formation volumes in $\mu$CT cross sections as was performed in Goff et al. (2012) and Matheny et al. (2013). Statistics regarding resorption cavity size and depth, formation fractions and activation frequencies are based on tens of thousands of remodelling events instead of a few dozen.

The objectives of this work are to:

1. Introduce a non-invasive method for studying tissue level consequences of growth and remodelling in trabecular bone through modelling.
2. Explain the spatio-temporal changes in trabecular bone density in various experimental conditions. Here, and throughout, bone density will refer to the amount of bone per unit tissue volume, denoted as BV/TV in the bone literature, as opposed to the mineral density. This objective will be stated more explicitly in Sect. 2.1 once the data and context of the study have been presented in more detail.
3. Determine, non-invasively, characteristics of individual remodelling events including resorption volume, resorption cavity depth, formation fraction and, at the tissue level, activation frequency.

Both models are calibrated by viewing the task of determining model parameters as an inverse problem. Thus the models are run with many combinations of candidate model parameters within a scheme for minimising the error between model results and observed data.

Details of the model are explained in the context of data from three experimental groups of growing female rats: (1) a control group, (2) rats having undergone ovariectomy and (3) rats having undergone ovariectomy and subsequent treatment with Zoledronic acid, a bishphosphonate. The data used here were obtained in the course of a previous study Fazzalari et al. (2012) but are briefly described in Sect. 2.1. The PDE model for bone density is introduced in Sect. 2.2 and an algorithm for simulating tissue level consequences of remodelling events is introduced in Sect. 2.3. Results of implementing the models appear in Sect. 3 followed by a discussion in Sect. 4.

## Model Details and Implementation

Although the models apply generally, details of implementation are specific to a particular setting. Here the models are introduced in the context of a study to explain, non-invasively, the patterns of changes in bone density resulting from growth and remodelling and determine the characteristics of remodelling in cancellous bone in the tibias of growing female rats.

All computations were performed using code written by the authors in Matlab.

### Data

**Fig. 1:**
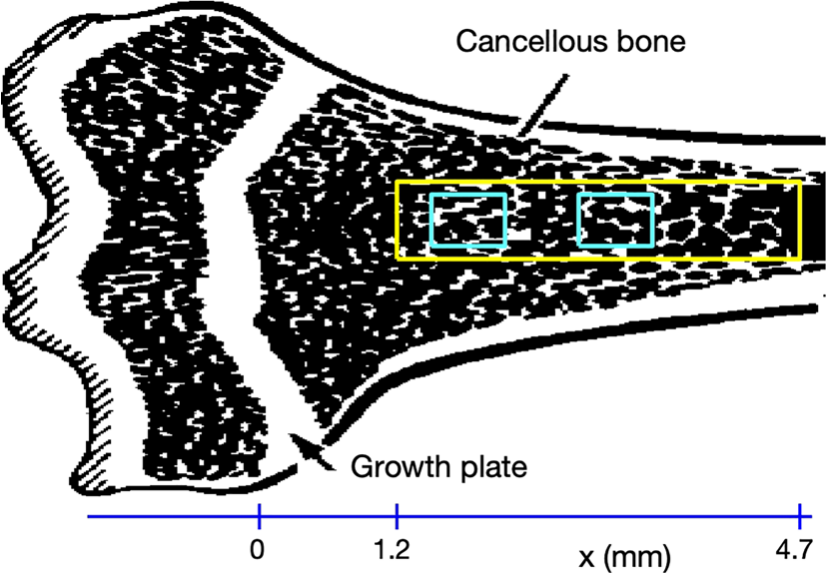
A sketch of the proximal end of a tibia showing the growth plate and the approximate location and size of the data block (yellow box) used in the study. The rats in the study were growing and so new bone emerging from the growth plate was moving away from the growth plate to the right. The two blue boxes within the yellow data block are data sub-blocks used to run remodelling simulations (Sect. 2.3). At time $t_1$, the sub-block is close to the left end of the data block but moves a distance $\Delta x = v(t_2-t_1)$ to a new position at time $t_2$. Here *v* is the rate at which new bone moves away from the growth plate. The sketch shows a 2D cross section, the data for this study was 3D (Color figure online)

**Fig. 2:**
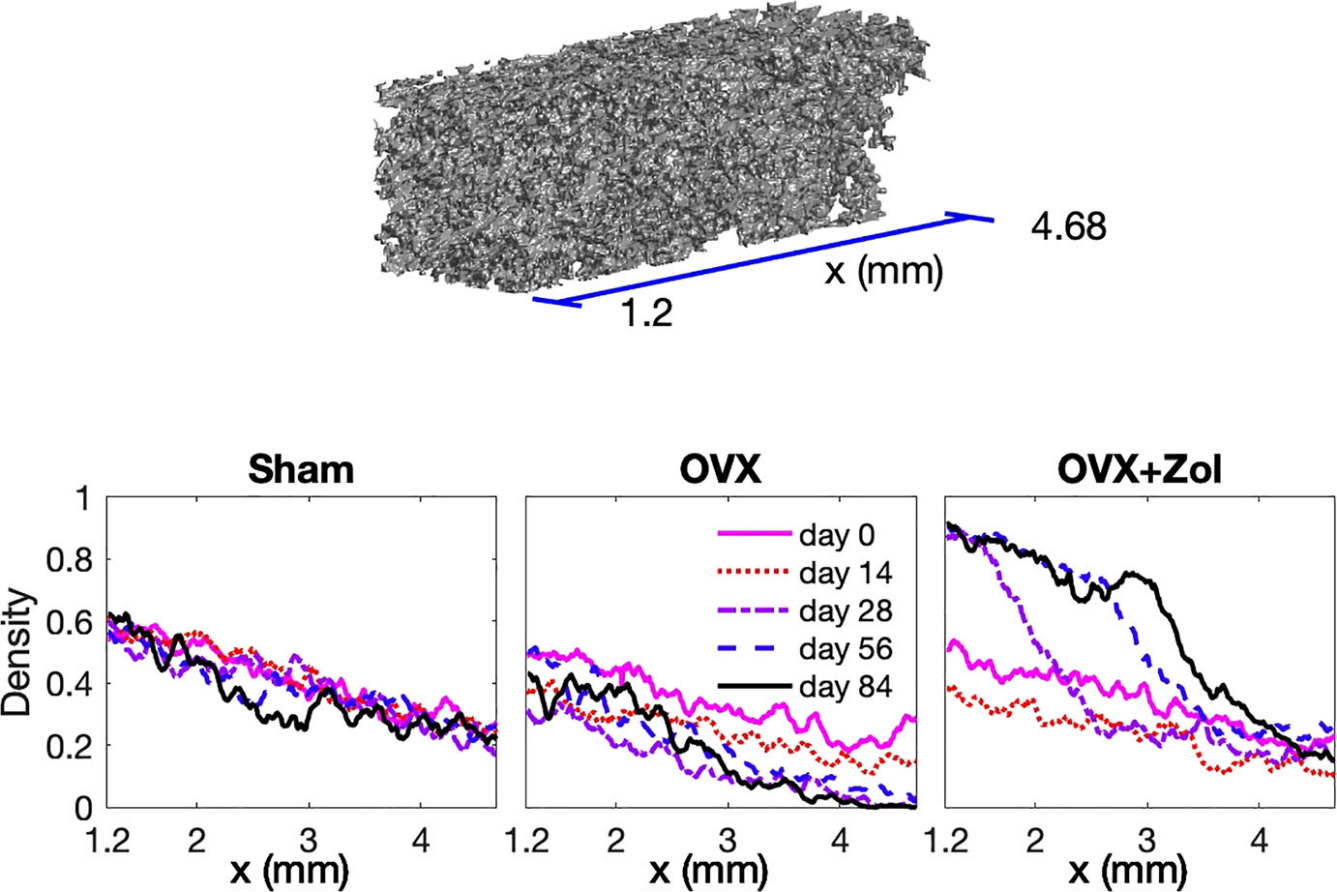
Data block and density plots. The top panel shows an example of a 3D data block from a $\mu$CT reconstruction of the region of a tibia indicated by the 2D sketch in Fig. 1. This example is from an OVX rat at day 14. The density of the trabecular bone is higher near the growth plate (near $x = 1.2$) than farther from the growth plate (near $x = 4.7$). Each of the three lower panels show plots of density, $T(x,t_j)$, as a function of *x* for a single rat selected from the indicated group at the five time points $t_i = 0, 14, 28, 56, 84$ (days). The horizontal axis is *x*, the distance from the growth plate, and matches the axis shown in the top panel in this figure and the *x*-axis in Fig. 1

The data used in this study were collected in the course of previous work and have been described in detail (Fazzalari et al. 2012; Perilli et al. 2010). Briefly, 30 juvenile female Sprague-Dawley rats aged eight weeks were randomly assigned to three experimental groups of 10 rats each. On day zero, the first group (Sham group) underwent sham ovariectomies (ovaries exposed but not removed), the second group (OVX group) underwent ovariectomy and the third group (OVX + Zol group) underwent ovariectomy but started treatment with Zoledronic acid on day 14 with weekly treatment up to day 77. In vivo $\mu$CT scans of the right tibia were performed on each rat at days 0, 14, 28, 56 and 84. Cubic voxels in the 3-dimensional reconstructions were of side length 8.7025 $\upmu$m. Three rats died in the course of the study and data were corrupted in one additional rat leaving complete data available for 10 Sham rats, 7 OVX rats and 9 OVX + Zol rats. For the current study, a rectangular data block of trabecular bone of size $121\times 121\times 400$ voxels (about $1\,\textrm{mm} \times 1\,\textrm{mm} \times 3.5\,\textrm{mm}$) was virtually extracted from each $\mu$CT scan (Supplementary material). Each data block was located within the trabecular region from about 1.2 to 4.7 mm from the growth plate (Fig. 1). An example of an actual 3D $\mu$-CT data block appears in Fig. 2.

For each rat and each time point $t = t^\star$, the projection of the 2-dimensional slice of the data block perpendicular to the axis of the bone at position *x* onto the axis of the bone was used to generate $T(x,t^\star )$, the true density of the data block as a function of *x* (Fig. 2).

Plots of $T(x,t^\star )$ as functions of *x* for fixed $t^*$ were consistent within experimental groups but distinct between groups. An example of $T(x,t^\star )$ for one rat from each experimental group at all five time points appears in Fig. 2. For the Sham rat, the density plots at the five different times overlay each other. The density is higher near the growth plate (left side of the figure) but this stays fixed over time. This suggests that the combined dynamics of bone growth and remodelling is stable: new bone emerging from the growth plate exactly matches the loss due to remodelling. For the OVX rat, the plots are qualitatively the same at every time point but are generally lower at successive time points. This system is not stable. New bone from the growth plate does not entirely replenish bone loss due to remodelling. The OVX + Zol rat follows the OVX regime during the first 14 days and so the plots for day 0 and day 14 are similar to the corresponding plots for OVX rats. However, starting at day 28 a vast increase in density is seen at the left end (near $x = 1.2$) of the window. This region of higher density moves to the right over time. Once this density front passes a point *x* on the horizontal axis, the curves once again overlay each other: the data plots for days 56 and 84 approximately coincide for $x \in [1.2,2.7]$ the data plots for days 28, 56 and 84 roughly coincide for $x\in [1.2,1.5]$. This indicates that, after treatment, a new equilibrium has been established.

Study objective 2 listed in Sect. 1.4 may now be stated more specifically as that of explaining the patterns of bone density over space and time for the three experimental groups in Fig. 2 quantitatively and, furthermore, to decide if growth and remodelling suffice to capture the essence of these patterns.

### PDE Model for Bone Density

Bone density will be denoted as *B*(*x*, *t*) where *x* is the distance from the growth plate along the axis of the bone (Fig. 1) and *t* is time. At time $t + \Delta t$ the bone at position *x* is has moved a distance $\Delta x = v\Delta t$ from the location at time *t*, where *v* is the rate at which bone moves away from the growth plate. During this time interval, the bone has also undergone remodelling. Hence

1 $$\begin{aligned} B(x,t+\Delta t) = B(x-v\Delta t,t) - S(t)B(x-v\Delta t,t)\Delta t, \end{aligned}$$

where *S*(*t*) is the rate of bone density loss due to remodelling. Writing (1) as a difference quotient and letting $\Delta t \rightarrow 0$ results in the first-order linear PDE

2 $$\begin{aligned} B_t + vB_x = -SB. \end{aligned}$$

To this point, the model is general. In the particular setting considered here, *t* is in days and *x* is in mm.

The model for the function *S* and the boundary conditions are specific to the application. Here, without further information available, *S* was modelled as piecewise constant with possible jumps at the time of ovariectomy and at the time of the start of treatment. Instantaneous jumps are not realistic biologically and preliminary inspection of the data confirmed gradual changes should be modelled. Accordingly, the rate of bone loss due to remodelling was modelled as

3 $$\begin{aligned} S(t)&= \frac{1}{2}(S_1 + S_3) + \frac{1}{2}(S_2 - S_1)\tanh (\lambda _1(t- J_1)) \nonumber \\&\quad + \frac{1}{2}(S_3- S_2)\tanh (\lambda _2(t - J_2)), \end{aligned}$$

where $S_i$, $i = 1,2,3$ are the constant rates of bone loss due to remodelling for the three treatment regimes: pre-ovariectomy, post ovariectomy but prior to treatment and post initiation of treatment, respectively. Here $J_1$ and $J_2$ are the times of ovariectomy and initiation of treatment respectively. Nominally, $J_1 = 0$ and $J_2 = 14$ (days) but these were left as parameters to be determined to account for any variation in times of day when ovariectomy or treatment was administered (exact times were not available) and any further natural metabolic delays. Parameters $\lambda _1$ and $\lambda _2$ reflect the speed at which the consequences of ovariectomy and treatment take effect and these were also treated as unknown.

The boundary condition was set at the location of the growth plate even though this lies about 1.2 mm to the left of the data window (Figs. 1 and 2). The density of bone emerging at the growth plate as a function of time, denoted *D*(*t*), was also modelled as piecewise constant but preliminary investigation showed no difference in this density between Sham and OVX regimes and so a jump was only modelled for the initiation of treatment. Again allowance was made for the exact time of treatment and the speed at which treatment takes effect. Hence the boundary condition at the growth plate was set as $B(0,t) = D(t)$, where

4 $$\begin{aligned} D(t) = \frac{1}{2}(D_2 + D_1) + \frac{1}{2}(D_2 - D_1)\tanh (\eta (t - K)). \end{aligned}$$

Here $D_1$ and $D_2$ are the constant densities of bone emerging from the growth plate before and after initiation of treatment respectively. The delay *K* could be different from $J_2$ but these two parameters were set equal for convenience.

The objective was to learn the model parameters from the data. For every rat at each of the five time points for which $\mu$CT scans were available (days 0, 14, 28, 56, 84) there are nominally 12 model parameters: the eleven parameters already introduced *v*, $S_1$, $S_2$, $S_3$, $\lambda _1$, $\lambda _2$, $J_1$, $J_2$, $D_1$, $D_2$, $\eta$ (recall *K* was set equal to $J_2$) plus a shift *w* along the axis of the bone included in the solution as $B(x-w,t)$. The shift parameter was needed to compensate for ambiguity in establishing the zero point where new bone emerges from the growth plate. The growth plate is not a rigid plane perpendicular to the axis of the bone (Fig. 1) which itself is not entirely straight either. Also, the growth plate is not infinitely thin. Instead, the view was taken that $x=0$ should represent some *effective* location where new bone forms. Only the seven parameters *v*, $S_1$, $S_2$, $\lambda _1$, $J_1$, $D_1$, *w* are needed for OVX rats since there is no change of condition due to initiation of treatment and only the four parameters *v*, $S_1$, $D_1$ and *w* play a role in Sham rats.

The error between the true bone density, $T(x,t^\star )$, and the modelled bone density, $B(x,t^\star )$ at a fixed time $t^\star$ was computed as

5 $$\begin{aligned} E = \sum _{i=1}^{400} \mid T(x_i,t^\star ) - B(x_i,t^\star ) \mid , \end{aligned}$$

where $x_i = 1.2 + (i-1)\delta$ and $\delta = 0.0087025$ mm. Here $\delta$ is voxel side length in the $\mu$CT reconstruction. The spacing in *x* was determined by the resolution of the data (Figs. 1 and 2).

Values of the model parameters were found by minimising *E* over the model parameters. All model parameters were viewed as fixed over all time points for a specific rat except the shift parameter *w* which was allowed to vary between time points since exact consistency of the location of the data block with respect to the growth plate could not be guaranteed from one $\mu$CT scan to the next due to small variations in positioning at image acquisition. The process of minimising *E* was hindered by the presence of numerous local minima, due, in part, to noise in the data.

To circumvent this difficulty, a ‘guided exhaustive search’ strategy was used as follows. For each parameter, a number of allowable values was set according information from the literature or by inspection of the data. For example, the densities $D_1$ and $D_2$ were initially restricted to the values $\{.4,.5,.6,.7,.8,.9\}$. Values for $J_1$, $J_2$ and *K* were initially fixed at 0, 14 and 14 (days) respectively since these reflect the known time of treatment. The model error was computed for all combinations of the allowable parameter values and the combination of parameter values with the smallest error was adopted as the current set of optimal parameter values. For the second stage, a new set of allowable values was constructed centred on the optimal set previously found but with a narrower range determined by the range of values found in initial stage and guided by visual inspection of model fits. This second criterion was necessary since local minima corresponding to poor model fits are possible due to noise. For $D_1$ the new set of allowable values was $\{.400,.475,.550,.625,.700\}$ while for $D_2$ the new values were $\{.85,.90,.95\}$. The new allowable values were $\{0, 1\}$ for $J_1$ and $\{14, 15\}$ for $J_2$ and *K*. In all, the model was run with approximately $1.2\times 10^8$ different combinations of parameter values for each individual rat.

This method for determining parameter values is generally not practical (and thus not popular) if there are very many parameters, if high precision is needed or if the forward model is time consuming to compute. In this case though, the number of parameters was reasonable, high resolution was not required and the forward model was very quick to compute because a closed form solution was available (“Appendix A”). The advantage was that data noise was less of an impediment.

### Simulation of Remodelling

Running a simulation required extracting two sub-blocks from each data block (Fig. 1): an initial sub-block on which to perform the remodelling simulations and a final sub-block for evaluating the results. Sub-blocks were of size $1.04\,\textrm{mm} \times 1.04\,\textrm{mm} \times 0.87\,\textrm{mm}$ ($120\times 120\times 100$ voxels). The initial sub-block was extracted from data block at time $t=t_1$ at a distance $x_1$ from the growth plate and the associated final sub-block was extracted from the data block at time $t= t_2$ at a distance $x_2 = x_1 + v(t_2-t_t)$ where *v* is the rate at which bone moves away from the growth plate (Fig. 1). Thus the final sub-block comprised the same patch of bone as the initial sub-block at a later time. Simulations of individual remodelling events were performed on the initial sub-block until the bone density matched that of the final sub-block.

Initial and final sub-block pairs were assigned to regime groups rather than experimental groups because rats in the OVX + Zol group were under the OVX regime until treatment started at day 14. Nominally, two sub-block pairs were extracted from each data block, the proximal pair closer to the growth plate ($x_1 \approx 1.6$ mm) and the distal pair farther from the growth plate ($x_1 \approx 2.5$ mm). Initial sub-blocks and final sub-blocks were taken from day 0 and day 14 respectively, from day 28 and 56 or from day 56 and day 84. For rats in the OVX treatment group, bone density was so low after day 56 that running simulations and measuring structure attributes was not feasible. Hence for OVX rats and, for consistency Sham rats, only sub-block pairs with initial sub-blocks from days 0 and 28 were used. For the OVX + Zol rats, this restriction severely limited the number of sub-block pairs because the pairs with initial sub-blocks from day 0 were allocated to the OVX regime. In addition, for all groups, some sub-block pairs could not be used because of insufficient bone density, particularly in distal pairs. Altogether, 36 sub-block pairs were extracted under the Sham regime, 44 under the OVX regime and 30 under the OVX + Zol regime (Table 1).

**Table 1:** Numbers of sub-block pairs according to regime and distance from the growth plate (proximal or distal)

Time	Proximal				Distal				Tot
	0–14	28–56	56–84	Tot	0–14	28–56	56–84	Tot	
Sham	10	10	–	20	7	9	–	16	36
OVX	16	7	–	23	16	5	–	21	44
OVX + Zol	–	9	9	18	–	5	7	12	30

In the second line, a–b refers to the time points (days) from which the initial sub-block was taken, a, and the time of the second sub-block, b

#### Simulating Individual Events

**Fig. 3:**
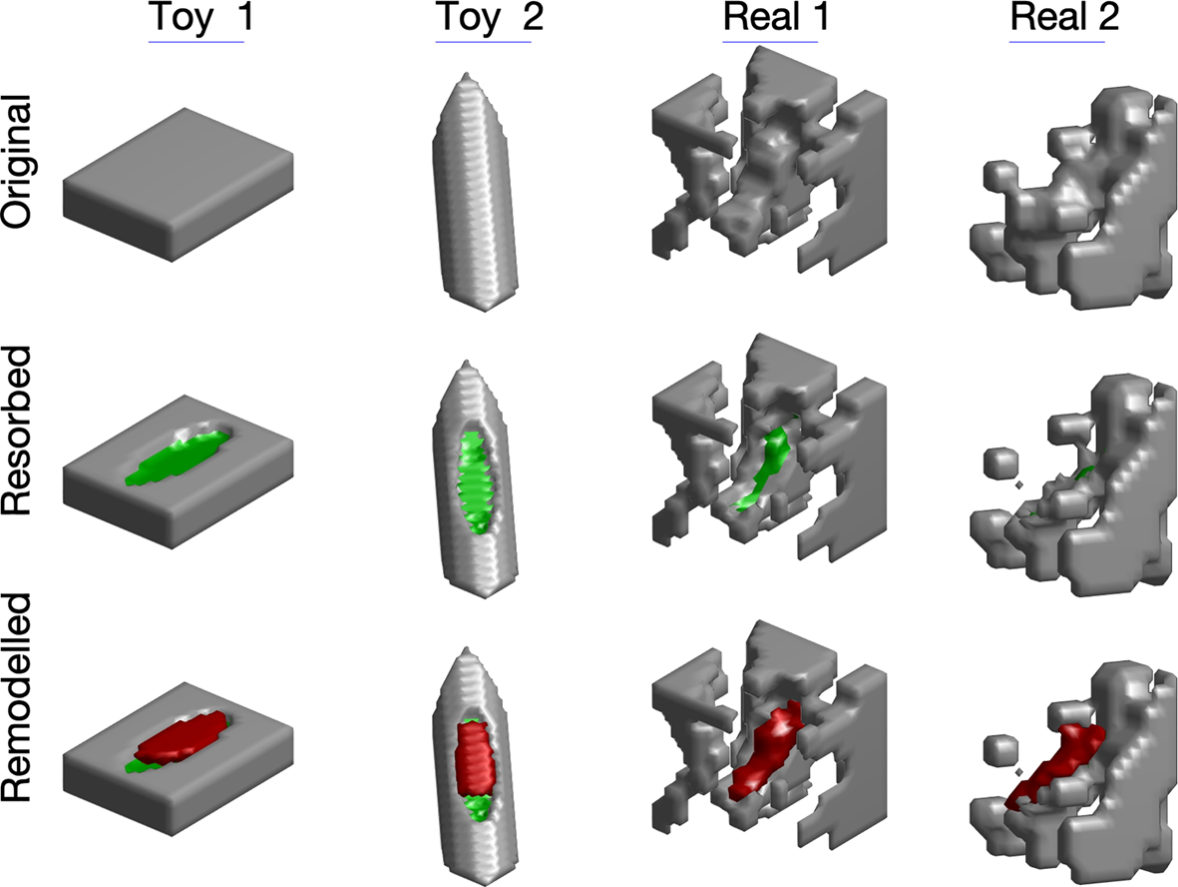
Examples of simulated remodelling events. Top row: original bone surface; middle row: after resorption; bottom row: after formation. The first two columns are toy examples and the second two columns are taken from $\mu$CT scans of rat tibia used in this study. The real examples are cubes of side length 182.7 $\upmu$m. Voxels comprising the newly exposed resorbed surface are displayed as green and the newly formed bone voxels are red for illustration purposes. In the toy examples, formation fraction is set to $\alpha = 0.8$. For the real examples, $\alpha = 1$. In the second real example, the viewing angle of the sample is such that most of the green surface is hidden. This angle was chosen to show that a portion of bone became disconnected during the resorption stage and, true to biological reality, was not reconnected during formation even though $\alpha = 1$

To simulate a single remodelling event, a location *p* on the surface of the bone was selected at random. Next, the local orientation of the bone was defined by listing the coordinates of all the bone voxels within a radius *r* of *p*. The principal component of this set of coordinates was taken to be the major axis of an ellipsoid with major axis half length *r* and both minor axes of half lengths *r*/3. The bone voxels within this ellipsoid lying within *h* voxels of the surface of the bone were removed to form the preliminary resorption cavity. By this criterion, the resulting resorption cavity may comprise several disjoint sets of voxels which seemed unlikely from a biological perspective. Accordingly, only the largest connected component was taken as the final resorption cavity. If the local bone surface constitutes a plane, the resorption cavity thus formed has an elliptic footprint with major and minor axis half lengths *r* and *r*/3 and of depth *h* (Fig. 3, column 1). Thus, for small *h*, the resorption volume is $R \approx \pi r^2h/3$. On real bone surfaces, the cavity follows the local topography of the bone (Fig. 3, columns 3 and 4) and the actual resorption volumes vary considerably (Table 3) even with the same input values *r* and *h*. The same is true for the formation fraction, $\alpha$. This issue will be discussed further in Sect. 3.2.

The voxel within the resorption cavity farthest from *p* was assigned as the point where formation was initiated. From here, voxels within the resorption cavity were sequentially included as newly formed bone according to proximity to the point where formation was initiated, subject to the criterion of giving priority to voxels closest to the surface of the resorption cavity (not the original surface of the bone). A key feature of this processes is that the resulting formation algorithm has no memory of the original surface of the bone. If structures become disconnected from the local bone during resorption, they are not necessarily reconnected during formation (Fig. 3, column 4). Formation continued until a pre-assigned volume of formation, $\alpha R$, was reached. Here *R* is the target resorption cavity volume $R = \pi r^2\,h/3$ and $\alpha$ is the formation fraction.

For each of the 110 sub-block pairs (Table 1), 72 simulations were run with parameter values in the ranges

$$\begin{aligned} r \in [43.5, 87.0]\, \mathrm{(mm)} , \quad h \in [8.7, 26.1]\, \mathrm{(mm)}, \quad \alpha \in [0.6, 0.9]. \end{aligned}$$

The ranges for the parameters were guided by existing estimates (Matheny et al. 2013) and the values of *h* were further restricted by the resolution of the $\mu$CT data. Setting $\alpha \ge 1$ resulted in unrealistic numbers of remodelling events and so was not pursued.

#### Comparing Bone Structure

In order to determine the most realistic simulation parameters, the results of each of the 72 simulations run on the initial sub-block of a sub-block pair was compared to the final sub-block of the pair. Comparisons were based on structure attributes of the bone. Historically, measures of bone structure were based on histomorphometry and include trabecular number (Tb.N), trabecular separation (Tb.Sp), trabecular thickness (Tb.Th), bone surface (BS), bone surface per unit tissue volume (BS/TV), bone surface per unit volume of bone (BS/BV) (Parfitt et al. 1987), trabecular bone pattern factor (TBPF) (Hahn et al. 1992), structural model index (SMI) (Hildebrand and Rüegsegger 1997), connectivity density (Conn.D) (Odgaard and Gundersen 1993) and Euler’s number (EUL) (Gundersen et al. 1993). More recently, measures have been suggested based directly on 3-dimensional data. These include fitting ellipsoids to local structure (Gontar et al. 2018; Doube 2015) and directly measuring local branching characteristics (Asiri et al. 2020).

The criterion for inclusion of structure attributes in this study was that the values should consistently distinguish between the structure of the initial and final sub-blocks of sub-block pairs. Based on previous work (Gontar et al. 2018; Asiri et al. 2020) and some preliminary tests, eight attributes were selected: BS, BS/BV, TBPF, SMI, EUL, ConnD, AveBR and StdBR. Here AveBR and StdBR are the mean and standard deviation of the number of branches per unit volume of bone. Branching characteristics depend on measuring the number of intersections of the bone structure with a sphere of radius $\rho$ centred at a point of interest. Asiri et al. (2020) a branching profile was obtained by considering several different radii at each point. Here, preliminary runs showed that using more than one value of the radius did not increase performance and so only means and standard deviations of branching at radius $\rho = 43.5$ mm were used. For each sub-block, 5000 sites within the bone were selected randomly with replacement for measuring branching. To remove bias due to numerical range, attribute values were normalised by subtracting the mean and dividing by the standard deviation computed over all initial and actual final sub-blocks ($n = 220$). Structure attributes for sub-blocks after simulation of remodelling were not included in computing these statistics.

The objective was to determine separate remodelling characteristics for the three regimes Sham, OVX, OVX + Zol and also to compare proximal and distal regions. Thus there were six sets of sub-block pairs, denoted here by $\Omega _j$, $j = 1,2,\dots ,6$. The number of sub-block pairs within the set $\Omega _j$, denoted by $N_j$, varied according the treatment group and whether the set comprised proximal or distal sub-block pairs (Table 1). For a fixed combination of *r*, *h*, $\alpha$, the error according to attribute *A* over all sub-block pairs in set $\Omega _j$ was computed as

6 $$\begin{aligned} E_{r,h,\alpha }(\Omega _j,A) = \frac{1}{N_j}\sum _{i = 1}^{N_j} \mid A(\textrm{Sim}_i) - A(\textrm{Fin}_i) \mid , \end{aligned}$$

where $A({\textrm{Sim}_\textrm{i}})$ is the value of attribute *A* for the initial sub-block after simulated remodelling, $A({\textrm{Fin}_\textrm{i}})$ is the value of attribute *A* on the final (true) sub-block of sub-block pair *i*. For each set $\Omega$ and each attribute *A*, the set optimal simulation parameters $r_{op}(\Omega ,A)$, $h_{op}(\Omega ,A)$, $\alpha _{op}(\Omega ,A)$ was the combination or *r*, *h* and $\alpha$ yielding the lowest value of $E_{r,h,\alpha }$. The final set of optimal simulation input parameters for $\Omega$ was taken to be the mean over the optimal values for each attribute. Thus $r_{op}(\Omega ) = \frac{1}{8}\sum _{j=1}^8 r_{op}(\Omega ,A_j)$ and similarly for $h_{op}(\Omega )$ and $\alpha _{op}(\Omega )$.

In evaluating the error, the structure attributes were measured only in an inner region of each sub-block so as not to include a margin of about 0.1 mm within the faces of the sub-block. This was necessary because the simulation of a single remodelling event required extracting a cube of size $2r+1\times 2r+1\times 2r+1$ voxels centred at the target voxel. Hence all remodelling simulations were centred at a distance at least $r+1$ voxels from the edge of the sub-block. Although voxels within the margin might be involved in simulated remodelling events, they were not exposed to the full range of remodelling effects.

## Results

The PDE model for bone density and the simulation model for remodelling are not entirely independent as further explained in Sect. 4 but the results of implementing the models are considered separately for convenience.

### PDE Model Results

**Fig. 4:**
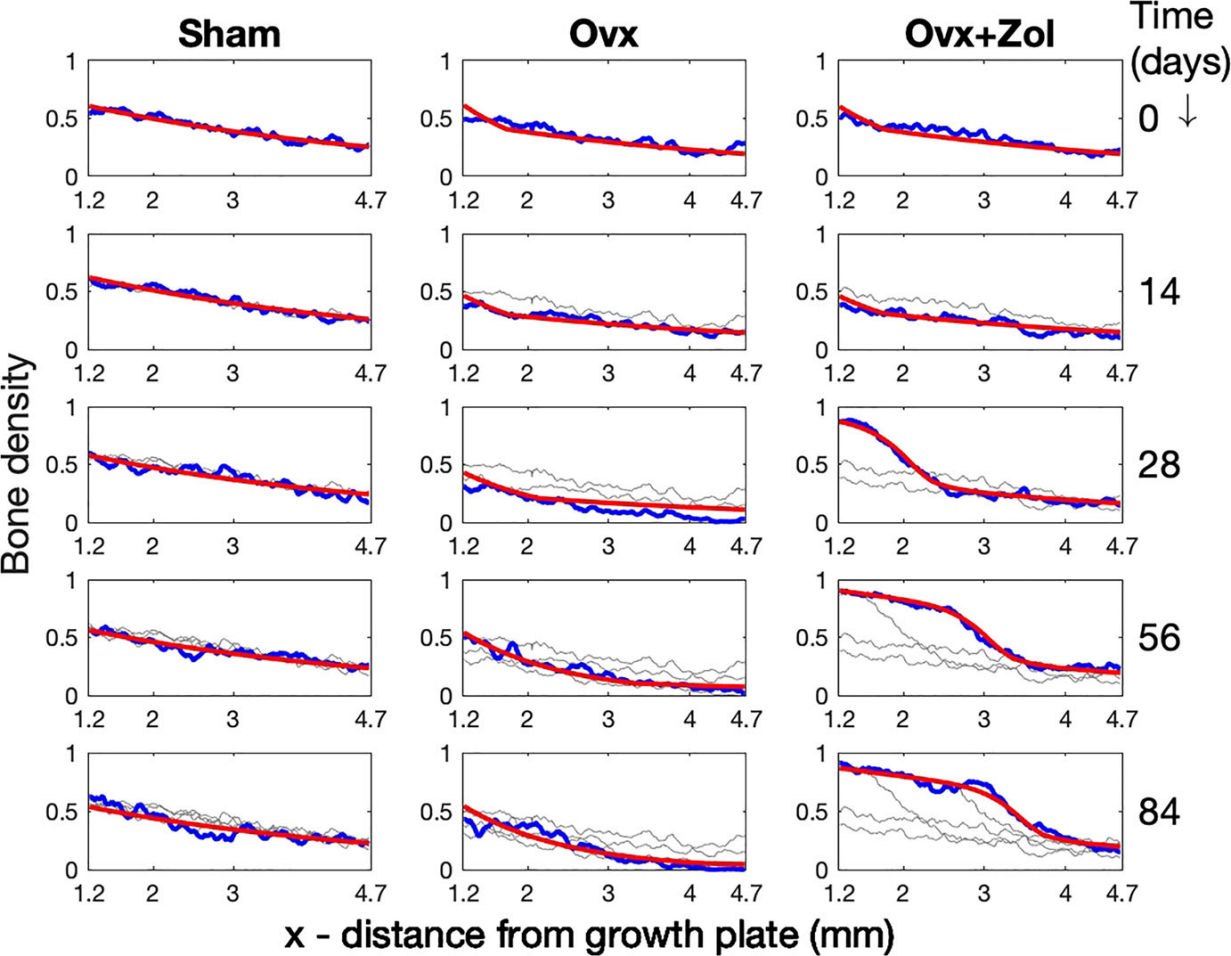
Plots of bone density data and model fits. Plots of bone density are shown for one rat from each of the three experimental groups at the five time points. Each column corresponds to a single rat chosen from the indicated experimental group and each row corresponds to a time point. In each panel, the horizontal axis is the distance *x* (mm) from the growth plate and the plots represent bone densities as a function of distance from the growth plate for the region within the data block as depicted in (Fig. 1), i.e. for $x \in [1.2,4.68]$. Thus each panel shows the spatial trend of bone density at a fixed time. The blue ragged plots show the true bone density data, $T(x,t^\star )$ and the red smooth plot shows the density predicted by the model, $B(x,t^\star )$ at the time point $t^*$ in days given on the right. In addition, the gray plots show true densities plots from previous time points to clarify the trends over time

The fits between the results of the PDE model and the observed data (Fig. 4) give confidence that the model captures the main patterns of bone density due to new bone emerging from the growth plate and remodelling.

Fitting the PDE model for bone density to the data involved finding values for 11 model parameters for OVX + Zol, six for OVX rats and three for Sham rats plus five shifts, one for each time point, for each rat in each experimental group. Values for all parameter resulting in the best fits appear in Appendix A. The focus here is on the density of new bone emerging from the growth plate before treatment ($D_1$) and after treatment ($D_2$) and *v*, the rate at which new bone moves away from the growth plate (Table 2).

**Table 2:** Density of new bone and rates at which new bone moves away from the growth plate

	Sham $n=10$	OVX $n=7$	OVX + Zol $n=9$
$D_1$	$0.565\pm 0.041$	$0.579\pm 0.067$	$0.539\pm 0.082$
$D_2$	–	–	$0.900\pm 0.056$
*v* (mm/day)	$0.027\pm 0.005$	$0.028\pm 0.009$	$0.025\pm 0.005$

Entries are mean ± SD. $D_1$ is the density before treatment and $D_2$ is the density after treatment. The rate *v* is in mm/day

There was no evidence of significant differences in $D_1$ between the three groups but there was a 67 percent increase between $D_1$ and $D_2$ for the OVX + Zol group. There was no significant difference in *v* between the three experimental groups (Table 2). Thus neither ovariectomy nor treatment with Zoldronic acid seems to influence the rate at which new bone moves away from the growth plate. Hansson et al. (1972) reported values for the rate of length increase of the proximal tibia in female rats at ages ranging from 20 days to 100 days. At the onset of our study, rats were 56 days old and 140 days at the end of the study. According to Hansson et al, the rate at 56 days is 0.161 mm/day and, using a best fit exponential to extrapolate the results by Hansson, the rate at 140 days is 0.016 mm/day. Hence the rate found here, $v \approx 0.027$ mm/day falls within the range suggested by Hansson.

The values for $D_1$ are consistent between experimental groups and the standard deviations are small compared to the difference between the mean values for $D_1$ and $D_2$ (Table 2). A similar pattern holds for $S_1$, $S_2$, $S_3$ (Table 8). The values for *v* are consistent between the experimental groups and the standard deviations are small. These patterns indicate that the models depend critically on the parameters and hence that parameter values leading to good fits are well determined.

### Simulation Results

The simulation input parameters *r*, $\alpha$ and *h* are not of primary interest. These parameters were used as input values to the simulation algorithm to guide target resorption volumes, formation fractions and depths. Due to the complex topography of real trabeculae (Fig. 3), actual values for these parameters realised in applying the algorithm varied substantially from the input simulation parameters. The measured parameter values are the ones of interest since they describe the changes in bone needed to mimic actual remodelling. To distinguish between the input simulation parameters *r*, $\alpha$ and *h* and the actual parameters realised in the course of the simulations, the latter are denoted RV for resorption volume, FF for formation fraction and CD for cavity depth.

Simulations were run with various combinations of input parameters as described in Sect. 2.3.1. For the simulation run resulting in the best match between the resulting simulated trabecular structure and the actual trabecular structure, the measured remodelling parameters RV, FF and CD were recorded for each individual remodelling event. In the course of running a single simulation event, other remodelling parameters may be extracted. In this study the surface area, SA, defined as the surface area of bone removed during the resorption phase of an individual remodelling event, was also recorded.

There were significant ($p < 0.0001$) differences in resorption volume between regime groups. There was a 22.5 percent increase in resorption volume under the pooled OVX regime compared to the pooled Sham regime which, subsequent to treatment, reduced back down to about 7.5 percent above the Sham regime (Table 3). There was a 2.0 percent decrease in resorption volume between proximal and distal locations under the Sham regime (Table 3) which is statistically significant ($p < 0.0001$). This difference is probably of no practical significance since it is small compared to the standard deviations which are around 12.5 percent. The statistical significance stems from the large values of *n*. There were no practical or statistically significant differences between proximal and distal locations under OVX or OVX + Zol regimes. The patterns seen in resorption volumes were mimicked by surface areas of individual remodelling events (Table 3).

All differences in formation fraction between proximal and distal locations were small as were differences between regimes (Table 3). Differences were statistically significant ($p < 0.0001$) in all cases, but they were small compared to standard deviations and are probably of no practical significance.

**Table 3:** Means and standard deviations (SD) of measured resorption volumes (RV) $\times 10^4\,\upmu \hbox {m}^3$, resorption surface areas (SA) $\times 10^3\,\upmu \hbox {m}^2$ and formation fractions (FF) computed over a total of *n* individual simulated remodelling events over all sub-blocks

	*n*	RV	SA	FF
*Sham*				
Prox	41,563	5.134	5.901	0.737
		(0.650)	(0.747)	(0.112)
Dist	37,144	5.029	5.780	0.733
		(0.630)	(0.724)	(0.125)
Comb	78,707	5.088	5.849	0.735
		(0.643)	(.739)	(0.118)
*OVX*				
Prox	98,565	6.235	7.166	0.718
		(0.906)	(1.042)	(0.146)
Dist	75,758	6.239	7.171	0.708
		(0.925)	(1.062)	(0.155)
Comb	174,323	6.237	7.169	0.714
		(0.914)	(1.051)	(0.150)
*OVX + Zol*				
Prox	44,208	5.473	6.291	0.737
		(0.761)	(0.875)	(0.107)
Dist	21,982	5.466	6.283	0.768
		(0.769)	(0.884)	(0.125)
Comb	66,191	5.471	6.282	0.747
		(0.764)	(0.878)	(0.114)

Activation frequency was computed as

7 $$\begin{aligned} Ac.f = \frac{N_E}{A\times \Delta t}, \end{aligned}$$

where $N_E$ is the number of simulated remodelling events needed to match the bone loss observed in the data over the time interval $\Delta t$ and *A* is the surface area of the bone within the data sub-block under consideration. Combined proximal and distal pairs within regimes showed a nearly three fold increase in activation frequency between Sham and OVX regimes (Table 4). The values returned to just below the Sham level after treatment. All these are strongly significant ($p < 0.0001$) and are in general agreement with the literature Han et al. (1997).

There was no difference in activation frequencies between proximal and distal locations under Sham regime ($p = 0.7256$), there was a moderately significant difference between proximal and distal locations under the OVX ($p = 0.0398$) regime, and a strongly significant difference between proximal and distal pairs under the OVX + Zol regime ($p < 0.0001$) (Table 4). These results suggest a trend in decreasing activation frequency as a function of distance from the growth plate which is amplified under the OVX regime and under the OVX + Zol regime. However, this interpretation is tenuous since the trend could be due to overall decrease in bone density (and surface area) in trabecular bone as a function of distance from the growth plate. Activation frequency reflects the number of remodelling events per unit surface and unit time. Thus an estimate of local surface area is required. Choices regarding the extent of the local structure for estimating this surface area could influence the results dramatically. Here the total surface area of bone within the sub-block was used.

**Table 4:** Mean and SD of activation frequencies (events/year)

	Sham		OVX		OVX + Zol	
	Prox	Dist	Prox	Dist	Prox	Dist
Mean	2.867	2.761	8.840	7.473	2.087	1.066
SD	0.859	0.934	2.788	1.011	0.420	0.207
*n*	20	16	23	21	18	12

Prox., Dist and *n* are as in Table 3

For every regime and location group, the resorption cavity depth was found to be CD $= 87.0\,\upmu$m, the side length of a single voxel in the $\mu$CT reconstruction. Thus the resolution of the scans was not adequate to resolve small changes in resorption cavity depth and sufficed only to discount two-fold differences or larger between groups.

## Discussion

The excellent fits between the solutions to the PDE and the observed bone density data (Fig. 4) demonstrate that the main spatio-temporal patterns of bone density observed in the data (Fig. 2) may be explained for all three experimental groups by the simple model proposed in Eqs. (2)–(4). Neither growth nor remodelling are able to explain the data on their own. If the density of new bone emerging from the growth plate alone could explain all the patterns in (Fig. 2), then good fits would have been found by varying $D_1$ and $D_2$ only and keeping *S* fixed. Instead good fits could only be found by setting different values of *S* (denoted $S_1$, $S_2$ and $S_3$), for the three treatment groups. Previous work Fazzalari et al. (2012) showed that remodelling alone could not explain these patterns. Accordingly, the PDE in Eqs. (2)–(4) represents a minimal explanatory model for capturing the patterns of bone density in Fig. 2. Thus objective 2 stated in Sect. 1.4 and refined at the end of Sect. 2.1 has answered in the affirmative: The PDE model in (2), (3), (4) is able to predict spatial and temporal patterns of bone density over in three experimental conditions and the simulation model shows that these patterns are achievable with a realistic model for remodelling. This confirms that growth and remodelling suffice to explain the observed patterns of bone density.

**Fig. 5:**
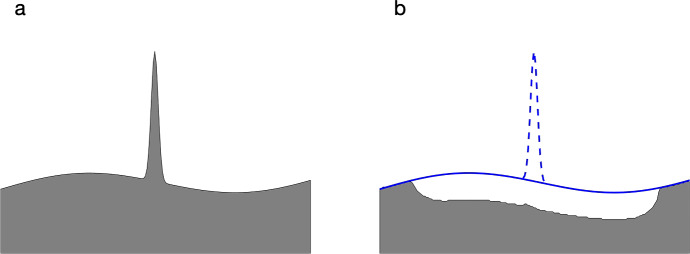
**a** Cross section of hypothetical bone. The large peak could be the cross section of a plate- or rod-like structure. **b** The same cross section after resorbing cells within distance *h* of the surface of the bone. The vertical structure is removed since it is thin compared to *h*. The solid line shows a boundary which might be assumed or estimated (using splines for example) if the resorption cavity is observed without knowledge of the structure prior to remodelling (which is always the case). The dashed line shows the unknown true original structure. The estimate of volume lost due to resorption based on the solid line underestimates the true resorption volume

Previous investigations reporting remodelling characteristics required manual identification and delineation of resorption events (Tkachenko et al. 2009). This process easily leads to bias in selecting sites and sizes of resorbed sites (Vanderoost and van Lenthe 2014). In these previous studies, the surfaces prior to resorption were not known and so had to be estimated, for example using splines (Goff et al. 2012; Matheny et al. 2013), potentially leading to substantial errors in values for resorption volumes and surface areas (Fig. 5). The process of identifying and measuring resorption cavities and formation in those studies was labour intensive and so statistics regarding remodelling characteristics were based on a few dozen events, or less, per group. The method presented here reports statistics based on tens of thousands of events per group. Resorption cavity volumes and surface areas are measured rather than estimated. Similarly, activation frequencies are based on the actual number of remodelling events and surface areas rather than extrapolations. But there is a tradeoff. A valid criticism of the method presented here is that these measurements, though precise, are made on simulated rather than actual remodelling events. The two main sources of potential error are that the models for resorption and formation do not incorporate the full biological complexity of remodelling and that the criteria for adopting values for the remodelling parameters *RV*, *CD* and *FF* based on matching bone structure may not capture the full scope of relevant structure attributes. Although no thorough study has yet been conducted, some preliminary runs were made during the development of this study with mild variations of the algorithms for resorption and formation (results not shown). These explorations indicated that the main patterns in remodelling characteristics reported in Tables 3 and 4 are robust against perturbations of the remodelling model. Previous work demonstrated that the structure attributes used here were successful in automatically classifying trabecular bone into Sham, OVX, and OVX + Zol groups (Gontar et al. 2018; Asiri et al. 2020) and so seem to capture key variation in structure between the groups.

In order to run the remodelling simulations, the data sub-blocks for each rat had to be tracked over time. One natural approach is to track trabecular structure directly. Boyd et al. (2006) proposed such a tracking method in 2026 but required the use of manually determined landmarks. Kemp et al. (2020) proposed direct tracking with exhaustive search using cross correlation as the cost function in 2020 and, in 2023, Ning et al. (2022) proposed direct rigid body registration as an initial step followed by gradient descent with mutual information as a cost function for final registration. The first approach by Boyd et al. was dismissed because of the need for manual intervention to set landmarks. A thorough study was conducted to implement the other two approaches (results not shown) but sub-blocks could not be tracked consistently in this manner, probably because the trabecular structure changed too much between time points in some rats to find reliable matches. Without direct tracking, matching sub-blocks at successive time points required information from the PDE model for bone density. In particular the shifts $\omega _j$ at time points $t_j$, $j = 1,2,3,4,5$ were needed to align the data blocks for an individual rat at the five time points. In addition, either *v*, the rate at which bone moves away from the growth plate, or the change in bone density expected between time points was needed to determine the location of a data sub-block at various time points. Both are available from the solution to the PDE model. Thus the model for simulating remodelling depended on the solutions to the PDE model.

Although the PDE model may be run on its own without input from the remodelling simulation model, the interpretation would not be satisfactory. The PDE model on its own does reproduce the bone density patterns seen in Fig. 2 but does not guarantee that these fits are achieved with realistic models for remodelling. For example, there is no way to know if the fits require unrealistic resorption cavity sizes or unrealistic numbers of events. The remodelling parameter *S* does not provide this information. To conclude that realistic remodelling suffices, the simulation model is required. In this sense, the models depend on each other.

In principle, the parameter *S* could provide a further link between the PDE and simulation models. However, *S* represent an average bone loss per unit time while the measured remodelling parameters *RV* and *FF* denote values for individual remodelling events. Hence the link is not of practical value.

The data and the modelling demonstrate that bone density varies greatly as a function of distance from the growth plate. This means that studies reporting a single value of bone density sampled within a band of the trabecular region may not reflect the full picture of bone density.

### Supplementary Information

Below is the link to the electronic supplementary material.

Supplementary file 1 (mat 343 KB)

Supplementary file 2 (mat 339 KB)

Supplementary file 3 (mat 333 KB)

Supplementary file 4 (mat 319 KB)

Supplementary file 5 (mat 321 KB)

Supplementary file 6 (mat 338 KB)

Supplementary file 7 (mat 270 KB)

Supplementary file 8 (mat 310 KB)

Supplementary file 9 (mat 272 KB)

Supplementary file 10 (mat 282 KB)

Supplementary file 11 (mat 298 KB)

Supplementary file 12 (mat 301 KB)

Supplementary file 13 (mat 298 KB)

Supplementary file 14 (mat 309 KB)

Supplementary file 15 (mat 281 KB)

Supplementary file 16 (mat 339 KB)

Supplementary file 17 (mat 341 KB)

Supplementary file 18 (mat 335 KB)

Supplementary file 19 (mat 305 KB)

Supplementary file 20 (mat 296 KB)

Supplementary file 21 (mat 348 KB)

Supplementary file 22 (mat 324 KB)

Supplementary file 23 (mat 316 KB)

Supplementary file 24 (mat 322 KB)

Supplementary file 25 (mat 316 KB)

Supplementary file 26 (mat 315 KB)

Supplementary file 27 (mat 317 KB)

Supplementary file 28 (mat 322 KB)

Supplementary file 29 (mat 283 KB)

Supplementary file 30 (mat 277 KB)

Supplementary file 31 (mat 331 KB)

Supplementary file 32 (mat 295 KB)

Supplementary file 33 (mat 240 KB)

Supplementary file 34 (mat 129 KB)

Supplementary file 35 (mat 70 KB)

Supplementary file 36 (mat 346 KB)

Supplementary file 37 (mat 336 KB)

Supplementary file 38 (mat 308 KB)

Supplementary file 39 (mat 306 KB)

Supplementary file 40 (mat 305 KB)

Supplementary file 41 (mat 319 KB)

Supplementary file 42 (mat 257 KB)

Supplementary file 43 (mat 157 KB)

Supplementary file 44 (mat 193 KB)

Supplementary file 45 (mat 178 KB)

Supplementary file 46 (mat 337 KB)

Supplementary file 47 (mat 349 KB)

Supplementary file 48 (mat 332 KB)

Supplementary file 49 (mat 312 KB)

Supplementary file 50 (mat 314 KB)

Supplementary file 51 (mat 355 KB)

Supplementary file 52 (mat 326 KB)

Supplementary file 53 (mat 223 KB)

Supplementary file 54 (mat 175 KB)

Supplementary file 55 (mat 149 KB)

Supplementary file 56 (mat 350 KB)

Supplementary file 57 (mat 295 KB)

Supplementary file 58 (mat 311 KB)

Supplementary file 59 (mat 319 KB)

Supplementary file 60 (mat 291 KB)

Supplementary file 61 (mat 343 KB)

Supplementary file 62 (mat 342 KB)

Supplementary file 63 (mat 329 KB)

Supplementary file 64 (mat 314 KB)

Supplementary file 65 (mat 320 KB)

Supplementary file 66 (mat 345 KB)

Supplementary file 67 (mat 303 KB)

Supplementary file 68 (mat 80 KB)

Supplementary file 69 (mat 66 KB)

Supplementary file 70 (mat 41 KB)

Supplementary file 71 (mat 337 KB)

Supplementary file 72 (mat 313 KB)

Supplementary file 73 (mat 305 KB)

Supplementary file 74 (mat 307 KB)

Supplementary file 75 (mat 299 KB)

Supplementary file 76 (mat 322 KB)

Supplementary file 77 (mat 247 KB)

Supplementary file 78 (mat 277 KB)

Supplementary file 79 (mat 310 KB)

Supplementary file 80 (mat 319 KB)

Supplementary file 81 (mat 291 KB)

Supplementary file 82 (mat 306 KB)

Supplementary file 83 (mat 309 KB)

Supplementary file 84 (mat 335 KB)

Supplementary file 85 (mat 329 KB)

Supplementary file 86 (mat 336 KB)

Supplementary file 87 (mat 338 KB)

Supplementary file 88 (mat 315 KB)

Supplementary file 89 (mat 322 KB)

Supplementary file 90 (mat 323 KB)

Supplementary file 91 (mat 342 KB)

Supplementary file 92 (mat 334 KB)

Supplementary file 93 (mat 315 KB)

Supplementary file 94 (mat 330 KB)

Supplementary file 95 (mat 322 KB)

Supplementary file 96 (mat 364 KB)

Supplementary file 97 (mat 312 KB)

Supplementary file 98 (mat 307 KB)

Supplementary file 99 (mat 280 KB)

Supplementary file 100 (mat 269 KB)

Supplementary file 101 (mat 298 KB)

Supplementary file 102 (mat 246 KB)

Supplementary file 103 (mat 288 KB)

Supplementary file 104 (mat 263 KB)

Supplementary file 105 (mat 269 KB)

Supplementary file 106 (mat 339 KB)

Supplementary file 107 (mat 339 KB)

Supplementary file 108 (mat 325 KB)

Supplementary file 109 (mat 308 KB)

Supplementary file 110 (mat 270 KB)

Supplementary file 111 (mat 341 KB)

Supplementary file 112 (mat 257 KB)

Supplementary file 113 (mat 184 KB)

Supplementary file 114 (mat 197 KB)

Supplementary file 115 (mat 94 KB)

Supplementary file 116 (mat 348 KB)

Supplementary file 117 (mat 333 KB)

Supplementary file 118 (mat 283 KB)

Supplementary file 119 (mat 191 KB)

Supplementary file 120 (mat 170 KB)

Supplementary file 121 (mat 331 KB)

Supplementary file 122 (mat 260 KB)

Supplementary file 123 (mat 207 KB)

Supplementary file 124 (mat 97 KB)

Supplementary file 125 (mat 103 KB)

Supplementary file 126 (mat 327 KB)

Supplementary file 127 (mat 345 KB)

Supplementary file 128 (mat 342 KB)

Supplementary file 129 (mat 324 KB)

Supplementary file 130 (mat 242 KB)

Supplementary file 131 (pdf 37 KB)

## Data Availability

Data comprising binarised 3-dimensional micro-ct reconstructions of rat tibias on which the experiments reported in this paper were based have been included as electronic supplementary material. A pdf file is included to explain content and file names. Briefly, the data files comprise 3D binary arrays with 1 indicating bone voxels. The files names are of the form ratXXwYY.mat where XX is a two digit integer to indicate the rat identification number ($0,1,\dots ,30$) and YY is a two digit integer to indicate the week number (0, 2, 4, 8, 12) corresponding to days 0, 14, 28, 56, 84. Files are stored in Matlab format and may be opened using the ‘load’ statement within Matlab. Experimental groups comprise rats according rat identification numbers as follows: Sham 1, 4, 6, 9, 11, 14, 17, 22, 23, 26; OVX 8, 10, 12, 15, 2, 28, 29. OVX + Zol 2, 5, 7, 13, 19, 21, 24, 25, 30.

## Appendix A: PDE Solutions

The PDE in (2), (3) with boundary condition (4) admits a closed form solution which may be found via the method of characteristics.

$$\begin{aligned} B(x,t) = D\left( t - \frac{x}{v}\right) e^{-Px}\frac{M_1(x,t)M_2(x,t)}{M_1(0,t)M_2(0,t)}, \end{aligned}$$

where

$$\begin{aligned} M_i(x,t) = \left[ 2\cosh \left( \lambda _i\left( t - \frac{x}{v} - J_i\right) \right) \right] ^{Q_i/\lambda _i}, \ i = 1,2 \end{aligned}$$

and

$$\begin{aligned} P = \frac{S_1+S_3}{2v},&Q_1 = \frac{S_2 - S_1}{2},&Q_2 = \frac{S_3 - S_2}{2}. \end{aligned}$$

For OVX rats, $S_3 = S_2$ (no treatment) and so $Q_2 = 0$ and hence $M_2 = 1$. For Sham rats, $S_3 = S_2 = S_1$ and so $M_1 = M_2 = 1$. In this case $P = S_1/v$.

## Appendix B: PDE model parameters

See Tables 5, 6, 7 and 8.

**Table 5:** Optimal parameter values found for the nine OVX + Zol rats

Rat Id	2	5	7	13	19	21	24	25	30
$D_1$	.475	.575	.500	.600	.400	.550	.650	.475	.625
$D_2$	.95	.90	.95	.85	.85	.80	.95	.90	.95
$\eta$	. 04	.10	.03	.04	.06	.03	.08	.04	.02
*v*	.032	.024	.020	.028	.028	.015	.028	.020	.028
$\lambda _1$	1.6	3.2	2.2	2.8	2.4	1.8	2.8	2.8	1.6
$\lambda _2$	.20	.05	.03	.05	.20	.04	0.2	.20	.20
$J_1$	1	1.5	2	1.5	1	1.5	1.5	0	1
$J_2$	15	16	16	16	14	15.5	16	15	14
$S_1$	.0085	.0030	.0030	.0050	.0070	.0050	.0050	.0075	.0070
$S_2$	.0200	.0160	.0100	.0300	.0200	.0120	.0400	.0200	.0200
$S_3$	.0010	.0040	.0020	.0070	.0030	.0050	.0030	.0050	.0010
$w_0$	0.8	0.2	0.1	0.1	0.6	0.1	0.2	0.4	0.2
$w_{14}$	0.2	0.1	0.3	0.1	0.2	0.1	0.1	0.2	0.2
$w_{28}$	0.8	0.5	0.4	0.2	0.4	0.1	0.1	0.4	1.0
$w_{56}$	1.0	0.5	0.8	0.1	0.6	0.1	0.5	0.4	0.4
$w_{84}$	0.2	0.2	0.2	0.3	0.2	0.3	0.2	0.2	1.0
Err	.0901	.1170	.0995	.1176	.0810	.1494	.1114	.1047	.1319

Parameter $w_t$ denotes the shift (mm) for the data at time *t* (in days). All other parameters were optimised over all time points simultaneously but separately for each rat. Units: $D_1$ and $D_2$ dimensionless, $\lambda _1$, $\lambda _2$, $\eta$, $S_1$, $S_2$ and $S_3$ in days^-1^, $J_1$ and $J_2$ in days

**Table 6:** Optimal parameter values found for the seven OVX rats

Rat Id	8	10	12	15	27	28	29
$D_1$	.600	.450	.600	.600	.600	.600	.600
*v*	.017	.035	.035	.017	.035	.035	.023
$\lambda _1$	0.4	3.6	3.6	3.2	2.8	0.4	1.6
$J_1$	2.0	0	0	0.5	1.0	2.0	2.0
$S_1$	.005	.009	.005	.003	.009	.005	.006
$S_2$	.030	.025	.030	.045	.040	.025	.035
$w_0$	$-$ 0.2	0.4	0.5	0	0.2	0.4	0.2
$w_{14}$	0	0	0	$-$ 0.2	$-$ 0.1	0.1	$-$ 0.1
$w_{28}$	0.1	$-$ 0.3	$-$ 0.1	$-$ 0.3	$-$ 0.2	$-$ 0.1	$-$ 0.2
$w_{56}$	$-$ 0.3	0.2	0	$-$ 0.3	0.2	$-$ 0.1	$-$ 0.3
$w_{84}$	$-$ 0.3	0.2	$-$ 0.2	$-$ 0.3	$-$ 0.3	$-$ 0.3	$-$ 0.3
Err	.0103	.1042	.1436	.1262	.1474	.1251	.1203

The units are as in Table 5

**Table 7:** Optimal parameter values found for the seven Sham rats. The units are as in Table 5

Rat Id	1	4	6	9	11	14	17	22	23	26
$D_1$	.600	.500	.600	.600	.600	.550	.550	.600	.550	.500
*v*	.032	.023	.032	.035	.029	.023	.020	.029	.020	.029
$S_1$	.003	.006	.007	.008	.006	.005	.005	.007	.004	.008
$w_0$	$-$ 0.3	$-$ 0.3	$-$ 0.3	$-$ 0.2	0	0.3	0.4	0.2	0.9	0.9
$w_{14}$	0.8	0.2	$-$ 0.3	0.3	0.8	1.0	0.5	0.5	1.1	1.3
$w_{28}$	1.1	0.4	0.4	$-$ 0.3	1.0	1.3	0.2	0.3	1.3	0.9
$w_{56}$	0.7	1.1	0.2	0.5	1.3	1.3	0.1	0.3	1.0	0.9
$w_{84}$	0.8	0.7	1.1	1.0	1.3	1.1	$-$ 0.1	1.1	0.4	0.2
Err	.1157	.0824	.0776	.0962	.1063	.0915	.0828	.0923	.0847	.1058

**Table 8:** Pooled values for $S_1$, $S_2$ and $S_3$

$S_1$ ($n = 26$)	$S_2$ ($n=16$)	$S_3$ ($n=9$)
$0.0058\pm 0.0019$	$0.0261\pm 0.0103$	$0.0034\pm 0.0020$

Values are grouped according to treatment regime rather than experimental group. $S_1$ applies to all 26 rats prior to ovariectomy and to Sham rats at all times. $S_2$ applies to rats having undergone ovariectomy but have not been treated with Zoledronic acid. This includes 16 rats: 7 OVX rats post ovariectomy and 9 OVX-Zol rats post ovariectomy but prior to treatment. $S_3$ only applies to the 9 OVX + Zol after treatment commenced. Values are mean ± SD
