# Supplementary material for: Modelling the Effects of Growth and Remodelling on the Density and Structure of Cancellous Bone
Source: Bull Math Biol. 2024 Mar 4;86(4):37. doi: 10.1007/s11538-024-01267-3 (PMC10912124; doi:10.1007/s11538-024-01267-3)
Supplement: Supplementary file 131 — (pdf 37 KB) [file 11538_2024_1267_MOESM131_ESM.pdf]

Data description

Initially 30 juvenile female Srague-Dawley rates aged eight weeks were assigned an identification number 1 - 30. The rats were assigned randomly to one of three experimental groups of 10 rats each: Sham, Ovx, Ovx+Zol.

On day zero, rats in the Sham group underwent sham ovariectomies: The rats were opened but the ovaries were left intact. On day zero, rats in the Ovx group and Ovx+Zol groups underwent ovariectomies. On day 14, rats in the Ovx+Zol group started weekly treatment with Zoledronic acid.

Three rats died in the course of the study and the data for another rat was corrupted. Accordingly, the study was based on 26 rats.

Table 1: Experimental group membership by rat identification number

Sham	1	4	6	9	11	14	17	22	23	26
Ovx	8	19	12	15	27	28	29			
Ovx+Zol	2	5	7	13	19	21	24	25	30	

Micro-computed tomography (μCT) scans were taken of the right tibia at days 0, 14, 28, 56, 84. The reconstructed μCT scans are in terms of cubic voxels of side length 8.7025μm. For this study, only a portion of the full reconstructions were used. A rectangular block $121 \times 121 \times 400$ voxels (approximately 1mm $\times$ 1mm $\times$ 3.5mm) located from between approximately 1.2 mm from the growth plate to 4.7 mm from the growth plate along the axis of the bone was extracted for each rat at each time point. Accordingly, the data consists of $5 \times 26 = 130$ rectangular binary blocks. Voxels with value 1 indicate bone and value 0 indicates non-bone.

The names of the files are of the form ratNNwMM.mat

NN is a two-digit number indicating the rat identification number in the range 1 - 30.
MM is a two-digit number indicating the week of the scan:
week 00 = day 0, week 02 = day 14, $\dots$, week 12 = day 84.

Thus the file rat13w04.mat is the reconstructed μCT scan of the right tibia of rat 13 at day 28.

The file format is .mat which may be read using Matlab or converted to other formats using various tools.

Please direct question regarding the data to Murk Bottema via email at the address murk.bottema flinders.edu.au
